# Preliminary Study on the *In Vitro* Antitumor Effects of *Nidus Vespae* on Gastric Cancer

**DOI:** 10.1155/2021/1549359

**Published:** 2021-06-07

**Authors:** Ming Zhu, Weiwei Han, Yang Ling, Qiufeng Qi, Yaping Zhang, Yun Peng, Lin Chen, Yanqing Bao, Yongping Liu

**Affiliations:** ^1^Clinical Oncology Laboratory, Changzhou Tumor Hospital Affiliated to Soochow University, Changzhou 213032, China; ^2^Central Laboratory, Changzhou Tumor Hospital Affiliated to Soochow University, Changzhou 213032, China; ^3^Emergency Department, Changzhou Tumor Hospital Affiliated to Soochow University, Changzhou 213032, China; ^4^Oncology Department, Changzhou Tumor Hospital Affiliated to Soochow University, Changzhou 213032, China; ^5^Medical School, Soochow University, Suzhou 215123, China

## Abstract

**Objective:**

The aim of this study was to investigate the *in vitro* antitumor effects of *Nidus Vespae* on gastric cancer and its ability to promote immune function.

**Methods:**

Cell viability was detected by the Cell Counting Kit-8 (CCK-8) assay. Cell cycle distribution and apoptosis were detected using flow cytometry. The THP-1 human monocytic cell line was used as a source of monocytic effector cells for analyzing proliferation and dendritic cell (DC) induction. Enzyme-linked immunosorbent assay was used to detect cytokine production, and multicolor flow cytometry was used to study the phenotype and functionality of THP-1 DCs.

**Results:**

A high concentration (>10 mg/mL) of *Nidus Vespae* decoction (NVD) inhibited SGC-7901 gastric cancer cell growth by inducing G2/M cell cycle arrest and apoptosis. However, a low concentration (≤10 mg/mL) of NVD significantly increased the proliferative ability of THP-1 in serum-containing medium and caused an increase in dendritic protrusions with the typical morphology of DCs compared to the negative control in serum-free medium. The THP-1 DCs had significantly increased expression of cluster of differentiation 11c (CD11c), CD40, CD80, CD83, and CD86, as well as secretion of tumor necrosis factor-alpha. Furthermore, the supernatant of THP-1 DCs significantly inhibited the proliferation of gastric cancer cells by inducing apoptosis and G1/S cell cycle arrest.

**Conclusions:**

Our findings suggest that NVD not only directly inhibits the growth of gastric cancer cells but also exerts indirect antitumor effects by enhancing immune function. These results provide an important theoretical basis for the clinical application of *Nidus Vespae* in gastric cancer treatment.

## 1. Introduction

Globally, gastric cancer remains a serious threat to human health given its high morbidity and mortality [1–3], and tumor treatment continues to be an important issue in modern medicine. With increasing globalization and advancements in modern medicine, novel therapeutic approaches such as targeted molecular therapy [4], immunotherapy [5], and latent/residual tumor cell ablation have been used to improve clinical outcomes for cancer patients, but the prognosis for patients with gastric cancer remains poor. An increasing number of patients receive complementary or alternative therapies, such as traditional Chinese medicine (TCM).

TCM has been practiced in China for more than 2,500 years and is becoming increasingly popular worldwide [6–8]. TCM takes a holistic approach; all parts of the human body are closely interconnected, and it is believed that the body is highly intelligent and can thus repair itself. When any part of the body changes, other parts show a corresponding reaction to maintain homeostasis. This theory is similar to the immune balance Western medicine concept. Western medicine previously focused mainly on the disease itself. However, with the development of immunology, western scientists are gradually realizing the importance of homeostasis in the human body, especially immune balance in tumor patients [9]. Imbalance of the immune system can lead to the development of tumors. In addition to the balance between tumors and the immune system, there is also a balance between tumors and the nonimmune cells that surround them [10]. If a therapeutic drug disrupts the balance between the tumor and healthy tissues, it may not exert antitumor effects; in fact, it may promote the progression of cancer. Thus, both the ability of antitumor drugs to kill tumor cells and their effects on immune function and normal tissue cells should be studied.

Recently, TCM has attracted increasing attention as an invaluable resource for the discovery of new drugs, with less toxicity and fewer side effects, which can improve immune function and quality of life [11, 12]. *Nidus Vespae* (the honeycomb of *Polistes olivaceus*, *Polistes japonicus* Saussure, and *Parapolybia varia* Fabricius) has been used for thousands of years in TCM to treat many diseases, such as rheumatoid arthritis, digestive and urinary disorders, dental diseases, and cancer [13, 14]. However, studies have focused on the antibacterial [15, 16] and anti-inflammatory effects [17] of *Nidus Vespae*-containing products; less attention has been paid to the role of *Nidus Vespae* in cancer treatment.

Our previous research found that *Nidus Vespae* decoction (NVD) has immune-enhancing effects on human peripheral blood immune cells *in vitro* [18]. In this study, we further explored the antitumor effects of crude extracts from *Nidus Vespae* on gastric cancer and immune cells.

## 2. Materials and Methods

### 2.1. Preparation of NVD

*Nidus Vespae* was extracted with boiling Milli-Q water (Millipore, Molsheim, France) for 30 min three times, followed by filtering, mixing of the decoction, and concentration by heating. In total, 25% of the crude extract was used as NVD, and the rest was purified by graded alcohol precipitation at final ethanol concentrations of 65%, 75%, and 85% to obtain NVD supernatant; after the alcohol evaporated, the solutions were designated as NVD65, NVD75, and NVD85, respectively. Then, all liquid preparations (NVD, NVD65, NVD75, and NVD85) were filtered through a 0.22 *μ*m syringe filter. Finally, a portion of each liquid preparation was used for the bacterial endotoxin test; the remainder was packed and stored at −80°C for later use.

### 2.2. Cell Culture

The SGC-7901 human gastric cancer cell line and THP-1 human monocytic cell line were purchased from the Stem Cell Bank of the Chinese Academy of Sciences (Shanghai, China). The SGC-7901 cells were maintained in RPMI 1640 medium (Invitrogen, Carlsbad, CA, USA) supplemented with 10% heat-inactivated fetal bovine serum (FBS; Gibco, Australia). The THP-1 cells were maintained in RPMI 1640 medium supplemented with 10% FBS and 0.05 mM *β*-Mer (Invitrogen). All cells were cultured in a humidified incubator at 37°C and 5% CO_2_.

### 2.3. Cell Proliferation Assay

SGC-7901 gastric cancer cells were treated with NVD, NVD65, NVD75, or NVD85 at concentrations of 10, 20, 40, and 80 mg/mL, and cell proliferation was determined by the Cell Counting Kit-8 assay (CCK-8; Dojindo, Kumamoto, Japan). Briefly, SGC-7901 cells (5 × 10^3^ cells/well) were seeded separately in 96-well plates and cultured in medium with or without NVD. Each group had three replicates and there was also a blank control. After 24, 48, and 72 h, 100 *μ*L fresh culture medium and 10 *μ*L CCK-8 solution were added to each well and incubated at 37°C for 1–4 h. The absorbance (*A*) was detected at 450 nm using a microplate reader (Bio-Rad, Hercules, CA, USA). The cell inhibition rate was calculated based on the background corrected absorbance using the following formula: inhibition rate (%) = (1−*A* experimental well/*A* untreated control well) × 100%, where *A* is the absorbance.

### 2.4. Stimulation of THP-1 by NVD

THP-1 cells (5 × 10^4^ cells/well) were incubated with different concentrations of NVD (0.312, 0.625, 1.25, 2.5, 5, 10, and 20 mg/mL; phosphate-buffered saline [PBS] was the control) with or without 10% FBS for 48 h. Morphological changes of THP-1 were observed, and cell proliferation was detected by the CCK-8 method using the following formula: proliferation rate (%) = *A* experimental well/*A* untreated control well × 100%, where *A* is the absorbance.

### 2.5. Enzyme-Linked Immunosorbent Assay (ELISA)

After treatment with 5 mg/mL NVD in serum-free medium for 48 h, the cell culture medium of THP-1 was collected and centrifuged at 10,000 rpm for 5 min, and the supernatant was centrifuged again at 4,000 × g to 200 *μ*L by ultrafiltration using the Amicon Ultra-4 Centrifugal Filter Unit (Millipore, Burlington, MA, USA) for further concentration. All procedures were performed at 4°C. The prepared supernatant samples were stored at −20°C and cytokine detection was performed within 1 week. The concentrations of inflammatory cytokines (interleukin-6 (IL-6), tumor necrosis factor-alpha (TNF-*α*), IL-12p40, interferon gamma (IFN-*γ*), and IL-10) were determined by ELISA (eBioscience, San Diego, CA, USA) according to the manufacturer's instructions.

### 2.6. Flow Cytometry Analysis of THP-1 Dendritic Cells (DCs)

THP-1 cells were cultured in serum-free medium containing 5 mg/mL NVD or PBS control for 48 h to induce THP-1 DCs. Then, the cultured cells were washed, suspended in cold fluorescence-activated cell sorting (FACS) solution, and stained with specific monoclonal antibodies (5 *μ*L/test) against cluster of differentiation 11c (CD11c), CD40, CD80, CD83, CD86, HLA-DR, or isotype controls (eBioscience) for 30 min. Then cells were washed twice, resuspended in 500 *μ*L cold FACS solution, and analyzed by flow cytometry (FACS Canton II; BD Biosciences, San Jose, CA, USA).

### 2.7. Endocytosis

NVD-stimulated THP-1 DCs were washed and resuspended at a concentration of 2.5 × 10^5^ cells/mL in culture medium containing 1 mg/mL FITC-labeled 40 kDa dextran (Sigma-Aldrich, St. Louis, MO, USA) and incubated for 10 min at 37°C. As a control, cells were incubated in the same medium or in medium without FITC-labeled 40 kDa dextran at 37°C. The cells were washed three times with cold PBS and analyzed by flow cytometry.

### 2.8. Cell Cycle and Apoptosis Detection

After treatment with 5 mg/mL NVD in serum-free medium for 24 h, the supernatants of THP-1 were collected for continued culture of SGC-7901 for 48 h, and the SGC-7901 cells were then collected and washed twice with cold PBS. We evaluated the SGC-7901 cell cycle with the propidium iodide (PI) staining cell cycle detection kit (Nanjing KeyGen Biotech, Jiangsu, China) and apoptosis induction with the annexin V-FITC/PI apoptosis detection kit (Nanjing KeyGen Biotech) using flow cytometry according to the manufacturer's instructions.

### 2.9. Statistical Analyses

All experiments were performed in triplicate and repeated three times. Data are shown as the mean ± standard deviation (SD). Means of multiple groups were compared using one- or two-way analysis of variance (ANOVA). The independent samples *t*-test was used to compare the means between two groups. The analyses were performed using GraphPad Prism software (GraphPad Software Inc., San Diego, CA, USA) and *P* < 0.05 was considered statistically significant.

## 3. Results

### 3.1. High Concentrations of NVD Inhibit Tumor Cell Growth

**Figure 1:**
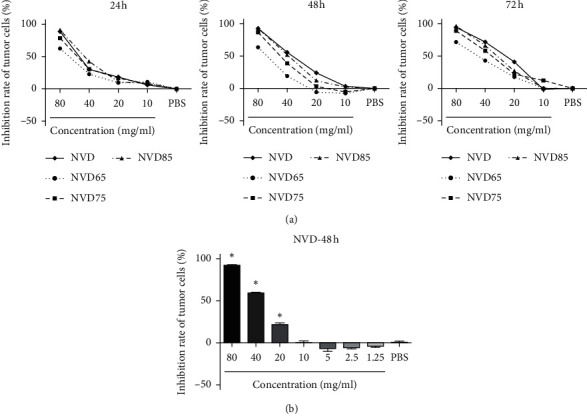
Inhibitory effects of different *Nidus Vespae* extracts on tumor cells. (a) Inhibition of SGC-7901 by treatment with different concentrations of NVD, NVD65, NVD75, and NVD85 (10, 20, 40, and 80 mg/mL) for 24, 48, and 72 h. Data was analyzed by two-way ANOVA with the Bonferroni post hoc test. (b) Inhibition rates of SGC-7901 after treatment with different concentrations of NVD (1.25, 2.5, 5, 10, 20, 40, and 80 *μ*g/mL) for 48 h. One representative result from three independent experiments with similar outcomes is shown. Data are presented as the mean ± SD and statistical significance was determined by one-way ANOVA followed by Dunnett's test (^*∗*^*P* < 0.05 compared to the PBS control).

The inhibition rate (%) of SGC-7901 was calculated after incubation with different concentrations of NVD, NVD65, NVD75, and NVD85 (10, 20, 40, and 80 mg/mL) for 24–72 h with PBS as the control. As shown in Figure 1(a), all four *Nidus Vespae* extracts significantly inhibited the growth of SGC-7901 cells in a concentration- and time-dependent manner (*P* < 0.05), and NVD had the strongest inhibitory effects. The inhibition rate markedly increased with the drug concentration (*P* < 0.05), and the 50% inhibitory concentration (IC_50_) of NVD on SGC-7901 at 48 h was about 36 mg/mL. However, when the concentration of NVD was less than 10 mg/mL, NVD showed weak proproliferative effects (Figure 1(b)). These results confirm that the regulatory effects of NVD (antiproliferative versus proproliferative) may differ by concentration. We took 10 mg/mL as the cutoff between high and low NVD concentrations and further studied their regulatory effects.

### 3.2. High Concentrations of NVD Affect the Cell Cycle and Apoptosis of Tumor Cells

**Figure 2:**
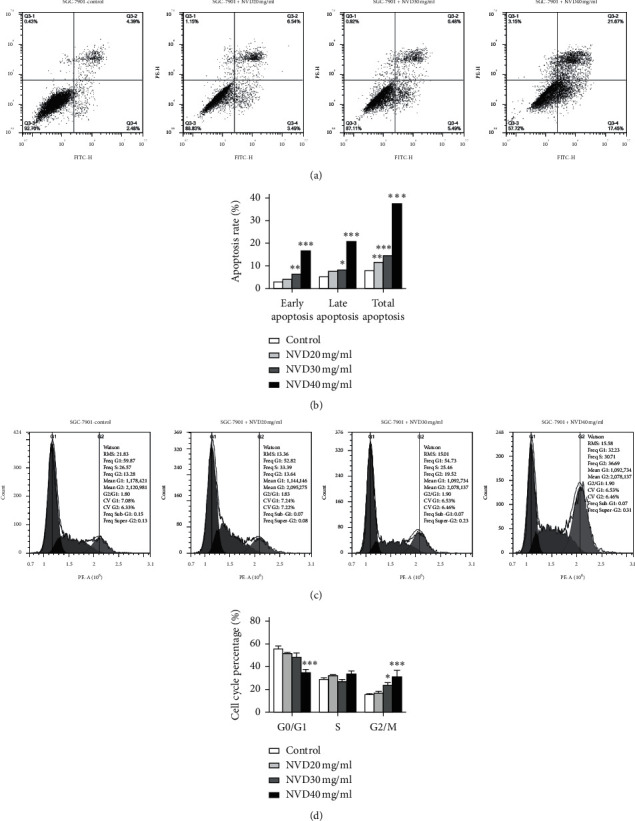
Effects of NVD on the apoptosis rate and cell cycle distribution in SGC-7901 cells. (a) Detection of apoptosis in SGC-7901 cells by flow cytometry. The cells were treated with various concentrations of NVD at 20, 30, and 40 mg/mL for 48 h and then stained with annexin V-FITC/PI and analyzed by flow cytometry. (b) The apoptosis rate of SGC-7901 cells in each group. The total apoptosis rate = percentage of early apoptosis + percentage of late apoptosis. (c) DNA content in SGC-7901 cells treated with various concentrations of NVD. After NVD treatment for 48 h, the cells were stained with PI and analyzed by flow cytometry. (d) Proportions of cells in each cell cycle stage based on the DNA content. The data are mean ± SD, *n* = 3. ^*∗*^*P* < 0.05, ^*∗∗*^*P* < 0.01, and ^*∗∗∗*^*P* < 0.001, compared to the control.

The effects of three concentrations of NVD near IC_50_ or 1/2 IC_50_ (20, 30, and 40 mg/mL) on the cell cycle and apoptosis of SGC-7901 cells were analyzed by flow cytometry. The results showed that the apoptosis rate of SGC-7901 cells in the 40 mg/mL NVD group was significantly higher than that in the control group (Figures 2(a) and 2(b)). In addition, 40 mg/mL NVD increased the amount of G2 DNA in SGC-7901 cells compared to the control, and the results indicated cell cycle arrest in the G2/M stage (Figures 2(c) and 2(d)).

### 3.3. Low Concentrations of NVD Stimulate THP-1 Cell Proliferation and Differentiation

**Figure 3:**
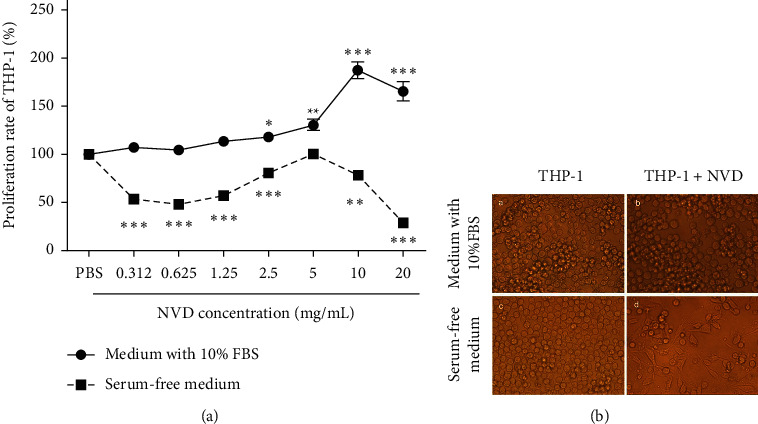
Low concentrations of NVD stimulate THP-1 proliferation and differentiation. (a) Proliferation rate (%) of THP-1 after NVD stimulation (0.312, 0.625, 1.250, 2.5, 5, 10, and 20 mg/mL) for 48 h. The solid and dashed lines represent culture of THP-1 in medium supplemented with 10% FBS and serum-free medium, respectively. The data are mean ± SD of three separate experiments using different batches of cells. All experiments were performed in triplicate. Data were analyzed by two-way ANOVA with the Bonferroni post hoc test (^*∗*^*P* < 0.05, ^*∗∗*^*P* < 0.01, and ^*∗∗∗*^*P* < 0.001, compared to PBS control). (b) THP-1 cells were cultured with 5 mg/mL NVD for 48 h and cell morphology was observed under a microscope. (A) THP-1 cells in medium supplemented with 10% FBS as the control; (B) THP-1 cultured with 5 *μ*g/mL NVD in medium supplemented with 10% FBS; (C) THP-1 in serum-free medium (control); (D) THP-1 cultured with 5 mg/mL NVD in serum-free medium.

To determine whether low concentrations of NVD promote the proliferation or differentiation of immune cells, the proliferation rate (%) and morphological characteristics of THP-1 cells were investigated during culturing with different concentrations of NVD (0.312, 0.625, 1.250, 2.5, 5, 10, and 20 mg/mL, with PBS as the control) in either serum-free medium or medium supplemented with 10% FBS. After 48 h culture in medium supplemented with 10% FBS, NVD caused a significant concentration-dependent increase in the number of THP-1 cells (*P* < 0.05; Figure 3(a)), although no significant change was observed in THP-1 cell appearance or shape (Figure 3(b)). In contrast, when cultured with NVD in serum-free medium, the proliferation activity of THP-1 cells generally decreased, except in the 5 mg/mL NVD group (*P* < 0.05; Figure 3(a)), and 5 mg/mL NVD induced obvious differentiation of THP-1 into DCs with characteristic stellate morphology (Figure 3(b)). These results suggest that NVD has different concentration-dependent effects on THP-1 cells under different culture conditions (serum-containing or serum-free medium).

### 3.4. Low Concentrations of NVD Enhance the Function of THP-1 Cells

**Figure 4:**
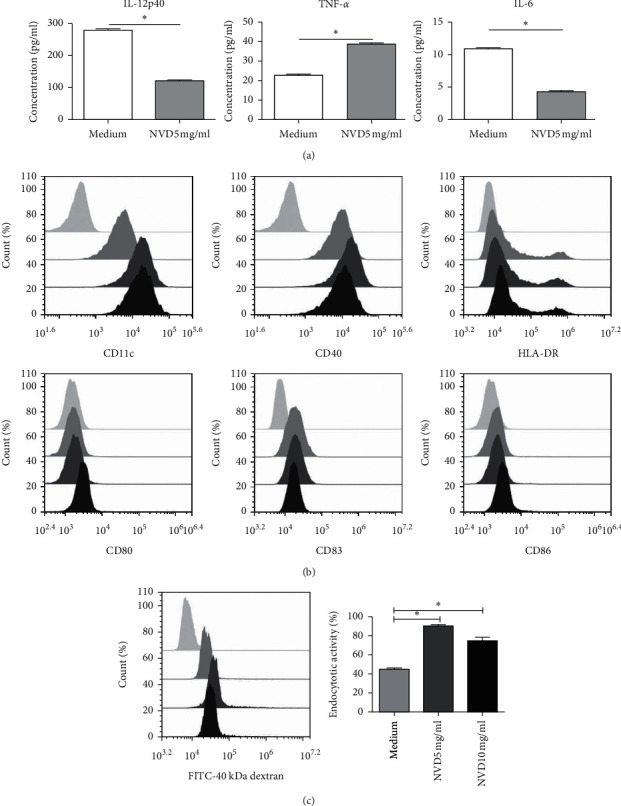
Effects of NVD on THP-1 function. (a) Secretion of proinflammatory cytokines induced by THP-1 after culturing with 5 mg/mL NVD in serum-free medium, as revealed by ELISA. (b) The expression of THP-1 DC surface markers was assessed by flow cytometry. The light gray peaks represent isotype control, the medium gray peaks represent medium only, the dark gray peaks represent 5 mg/mL NVD, and the dark peaks represent 10 mg/mL NVD. (c) Flow cytometry analysis of the endocytotic activity of THP-1 DCs. Uptake of 40 kDa dextran was measured after 10 min. Light gray peak: autofluorescence of THP-1 cells not incubated with 40 kDa dextran. Medium gray peak: control; dark gray peak: 5 mg/mL NVD; black peak: 10 mg/mL NVD. A histogram representing three independent experiments with similar results is shown. ^*∗*^*P* < 0.05.

We investigated the effects of NVD on immune cell function. First, to assess the effects of NVD on cytokine production by NVD-stimulated THP-1 cells, we collected and concentrated the cell culture medium and analyzed the secretion of inflammation-related cytokines by ELISA. As shown in Figure 4(a), NVD-stimulated THP-1 DCs secreted more TNF-*α*, but less IL-6 and IL-12p40, than controls (*P* < 0.05). There was minimal secretion of IFN-*γ* or IL-10 in both the experimental and control groups. Next, we used flow cytometry to detect surface markers of THP-1 DCs after culturing for 48 h in serum-free medium supplemented with 5 and 10 mg/mL NVD. As shown in Figure 4(b), 5 mg/mL NVD significantly upregulated the surface markers CD11c and CD40 (*P* < 0.05). Although the peaks for CD80, CD83, and CD86 shifted by only a small amount, the proportion of cells positive for THP-1 cell surface markers was increased (Table 1). Finally, to further characterize the DCs induced by THP-1, we determined their capacity for receptor-mediated endocytosis using FITC-labeled 40 kDa dextran. As shown in Figure 4(c), after culturing with 5 and 10 mg/mL NVD, THP-1 DCs displayed higher endocytotic activity than controls (*P* < 0.05). These data suggest that NVD may contribute to THP-1 cell differentiation.

### 3.5. Inhibitory Effects of the THP-1 Supernatant on Tumor Cells

**Figure 5:**
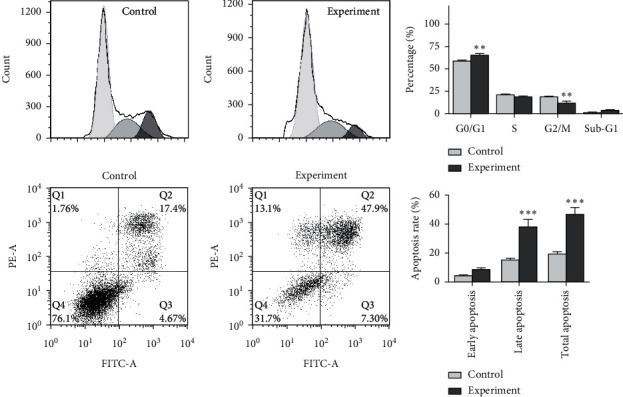
Effects of NVD-stimulated THP-1 supernatant on the cell cycle and apoptosis of SGC-7901 gastric cancer cells. Experimental group: 5 mg/mL NVD + THP-1 supernatant; control group: THP-1 supernatant. Data were analyzed by two-way ANOVA followed by the Bonferroni post hoc test. ^*∗∗*^*P* < 0.01 and ^*∗∗∗*^*P* < 0.001 compared to the control.

To examine the cytotoxic effects of NVD on tumor cells, the THP-1 cells were cultured with 5 mg/mL NVD or PBS control for 24 h, and the supernatant was collected for culture of the gastric tumor cell lines KATO-III, SGC-7901, and NCI-N87 for 48 h. We then analyzed cancer cells proliferation with the CCK-8 assay, as well as the cancer cell cycle and apoptosis by flow cytometry. The tumor cell inhibition rates for KATO-III, SGC-7901, and NCI-N87 cells in the experimental group (5 mg/mL NVD + THP-1 supernatant) are shown in Table 2 and were significantly higher than those in the negative control group (THP-1 supernatant; *P* < 0.05). Next, we analyzed the cell cycle by flow cytometry. The supernatant from the experimental group increased the amount of sub-G1 and G0/G1 DNA in SGC-7901 cells compared to the control (*P* < 0.05; Figure 5), indicating cell cycle arrest at the G1/S stage. The total apoptosis rate in the SGC-7901 cells in the experimental group was 55.2%, which was significantly higher than that of the control group (22.07%; *P* < 0.05; Figure 5).

## 4. Discussion

*Nidus Vespae* has been used in TCM for thousands of years to treat many diseases, including rheumatoid arthritis, digestive and urinary disorders, dental diseases, and cancer [14, 19, 20]. *Nidus Vespae* has strong anti-inflammatory and antibacterial effects [15, 16, 21] and also exerts antitumor [22–24] and immunomodulatory effects [18, 25]. Many studies have confirmed that tumorigenesis and development are closely related to immunity and inflammation, and we speculate that *Nidus Vespae* may have multiple beneficial effects in the context of tumor treatment.

In this study, we found that four kinds of *Nidus Vespae* extracts (NVD, NVD65, NVD75, and NVD85) significantly inhibited the growth of SGC-7901 cells, with NVD showing the strongest inhibitory effect. Our data suggest that water-alcohol extracts (NVD65, NVD75, and NVD85) may lose some of their active ingredients after ethanol precipitation from NVD. We also found that the inhibitory effects of NVD were only strong at high concentrations (>10 mg/mL). At low concentrations (<10 mg/mL), weak proproliferative effects of NVD were observed. Therefore, we speculate that the regulatory effect of NVD may differ by concentration: there may be cell cytotoxicity at high concentrations but growth-promoting effects at low concentrations.

Inducing apoptosis using TCM is an important strategy for treating cancer. Annexin V/PI showed that the apoptosis rate of SGC-7901 cells was positively correlated with the concentration of NVD. This indicates that the dose-dependent inhibitory effect of NVD on SGC-7901 cells is likely related to the induction of tumor cell apoptosis. PI staining showed that the number of cells in the G2/M stage was increased by NVC treatment, suggesting that the cell cycle was arrested. Research on the antitumor effects of *Nidus Vespae* has mainly focused on *Nidus Vespae* protein [22, 23, 26]. However, proteins are often inactivated during the high-temperature cooking process, so there will likely be other high-temperature resistant anticancer active ingredients in NVD. We analyzed the composition of NVD by liquid chromatography/mass spectrometry and gas chromatography/mass spectrometry; 56 compounds were isolated, mainly consisting of ketones, aldehydes, alcohols, and small amounts of rhein. However, to determine the compounds that exerted antitumor effects, further investigation is needed. Another possibility is that the various components in NVD exert their anticancer effects in combination. Because the clinical effects of TCM usually depend on the multiple components, quantification of one or even several active ingredient(s) is not necessarily sufficient for full characterization of the chemical properties. We found that the antitumor effects of NVD were stronger than those of water-alcohol extracts (NVD65, NVD75, and NVD85), which supports the above conjecture.

We also found that different concentrations of NVD have different effects. As mentioned above, high concentrations of NVD have a direct inhibitory effect on tumor cells; however, we also found that low concentrations of NVD can promote the proliferation or differentiation of THP-1 human monocytic cells. In serum-containing medium, NVD caused a significant concentration-dependent increase in the number of THP-1 cells, without any significant changes in cell appearance or shape. By contrast, in serum-free medium, although NVD decreased the proliferation activity of THP-1 cells, it clearly induced the differentiation of THP-1 cells into DCs with a characteristic stellate morphology. In addition, not only did 5 mg/mL NVD fail to suppress the proliferation of THP-1 cells, but also it had a greater effect on differentiation than any other treatment. The results suggested that the regulatory effects of NVD on THP-1 cells differ according to the culture conditions (serum-containing or serum-free medium), although the underlying mechanisms remain to be further explored.

THP-1 human monocytic cells can be induced to differentiate into macrophages by phorbol myristate acetate and then be converted into foam cells by exposure to oxidized low-density lipoprotein or differentiated into mature DCs [27] when cultured in serum-free medium containing GM-CSF, TNF-*α*, and ionomycin. We found that 5 mg/mL NVD can stimulate THP-1 to secrete more TNF-*α* than the control, and this resulted in significant upregulation of the surface markers CD11c and CD40. Although the peaks of CD80, CD83, and CD86 shifted by a small amount, the proportion of cells positive for THP-1 cell surface markers was increased. Moreover, THP-1 cells displayed greater endocytotic activity after culturing with 5 mg/mL NVD than the controls. These data suggest that NVD may stimulate THP-1 cells to differentiate into DCs. Furthermore, the supernatant collected from 5 mg/mL NVD-stimulated THP-1 significantly inhibited gastric tumor cell proliferation, arrested the cell cycle of SGC-7901 cells in the G1/S stage, and induced apoptosis in those cells. These results confirmed [18] that a low concentration of NVD is effective for promoting immune function.

In summary, we demonstrated that NVD not only directly inhibits gastric cancer cell growth but also exerts indirect antitumor effects by enhancing immune function. Our study provides an important theoretical basis for the clinical application of *Nidus Vespae* in gastric cancer treatment; however, the underlying mechanisms and signaling pathways of *Nidus Vespae* remain unclear. Given its potential for wide clinical application, *Nidus Vespae* merits further study.

## Figures and Tables

**Table 1:** Mean percentage of cells positive for THP-1 cell surface markers after culturing with NVD for 48 h.

Key	Treatment	Percentages of cells expressing surface markers (mean ± SD, *n* = 3)					
		CD11c	CD40	HLA-DR	CD80	CD83	CD86
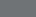	Control	10.20 ± 1.79	28.20 ± 1.42	18.94 ± 1.42	2.44 ± 0.69	0.85 ± 0.27	1.32 ± 0.46
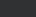	5 mg/mL NVD	59.48 ± 5.01^*∗∗∗*^	59.83 ± 2.83^*∗∗∗*^	23.70 ± 2.26	4.44 ± 0.94	4.45 ± 0.49^*∗∗*^	1.96 ± 0.38
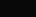	10 mg/mL NVD	54.85 ± 7.18^*∗∗*^	42.59 ± 1.71^*∗∗*^	22.51 ± 1.26	7.21 ± 1.14^*∗*^	4.20 ± 0.31^*∗∗*^	4.07 ± 0.37^*∗∗*^

^*∗*^ *P* < 0.05, ^*∗∗*^*P* < 0.01, and ^*∗∗∗*^*P* < 0.001 compared to the control.

**Table 2:** Inhibition rate of gastric tumor cells in different culture supernatants.

	THP-1 supernatant (control)	5 mg/mL NVD + THP-1 supernatant	*P* value
KATO-III	8.893 ± 3.154	27.29 ± 4.971	0.0354^∗^
SGC-7901	7.632 ± 3.486	36.01 ± 3.544	0.0047^∗∗^
NCI-N87	11.28 ± 2.800	32.80 ± 4.317	0.0139^*∗*^

^*∗*^ *P* < 0.05 and ^*∗∗*^*P* < 0.01 compared to the control.

## Data Availability

The data used to support the findings of this study are available from the corresponding author upon request.
